# HER2 expression should be routinely evaluated in DCIS to avoid under or overtreatment!

**DOI:** 10.18632/oncoscience.572

**Published:** 2023-01-30

**Authors:** Nidhi Garg, Mangesh A. Thorat

**Keywords:** DCIS, HER2, prognostic biomarker, predictive biomarker, radiotherapy

HER2 is more frequently overexpressed in ductal carcinoma *in situ* (DCIS) than in invasive breast cancer. However, unlike invasive cancer, HER2 is not routinely evaluated in DCIS. In the largest biomarker study in a DCIS randomized trial [1], we showed that HER2 overexpression was associated with a 2-fold higher ipsilateral breast event (IBE) risk mediated through almost 3-fold higher ipsilateral *in situ* event (DCIS-IBE) risk [Hazard ratio (HR) = 2.90; 95% CI, 1.91–4.40; *p* < 0.0001]. However, HER2 overexpression was also associated with a greater radiotherapy benefit. Adjuvant radiotherapy reduced ipsilateral *in situ* events by 84% in HER2-positive DCIS as compared with 42% reduction in HER2-negative DCIS (*P*_heterogeneity_ *=* 0.04). Ipsilateral invasive event (I-IBE) risk, although higher [HR = 1.40; 95% CI, 0.81–2.42; *P* = 0.23], was not significantly elevated in HER2-positive DCIS.

In this comment, we discuss the significance and clinical implications of these results. We also hope to convince the reader that more robust data are unlikely to be available for a foreseeable future and therefore clinical practice needs to change based on these results and start routinely evaluating HER2 to prevent under or overtreatment of DCIS patients.

The UK/ANZ DCIS biomarker study is based on a randomised trial with a mature follow-up and therefore not prone to suffer treatment-related confounding (below). Additional careful design measures like nested case-control sensitivity analyses further eliminated the possibility of residual treatment-related confounding. These results can help resolve ambiguities in implementing adjuvant treatment guidelines (e.g. ESMO). For example, widely prevalent radiotherapy overtreatment can be eliminated in low/intermediate grade DCIS <10 mm when it does not overexpress HER2. On the other hand, in a large DCIS lesion where a complex oncoplasty procedure is required, mastectomy with immediate breast reconstruction may be more appropriate if such DCIS overexpresses HER2. This will eliminate the need for adjuvant radiotherapy and associated harms especially if it is a left sided disease.

These results also provide new mechanistic insights. They point to HER2 overexpression being an early event in DCIS development, but with a limited role in progression to invasive disease. It can be hypothesised that being an early event, HER2 overexpression is more widespread in the breast and thereby predisposes the breast to develop new DCIS lesions that manifest as *in situ* events. This hypothesis is consistent with radiotherapy benefit being accrued through eradication of these potential foci. Furthermore, the preliminary findings from the NSABP-B43 trial [2] in HER2-positive DCIS are also entirely consistent with this hypothesis. Two-thirds of all events in control arm of this trial were *in situ* events and HER2-blockade by two doses of trastuzumab resulted in a nonsignificant 32% reduction (HR = 0.68; 95% CI, 0.43–1.08; *P* = 0.10) in the DCIS-IBE risk whereas the risk of I-IBE was not altered (HR = 1.11; 95% CI, 0.59–2.10; *P* = 0.74).

Prior to our study [1], just about a third of studies (10 of 27) investigating the association between HER2 overexpression and recurrence risk reported a significant association. While lack of power may have contributed to the observation of a null association in many of these studies, the role of treatment-related confounding unfortunately remains largely underappreciated. We shall explain treatment-related confounding with HER2 as an example (see Table 1).

**Table 1:** Effect of treatment-related confounding with or without predictive interaction on sample size for an adequately powered study

Predictive interaction	Subgroup	Treatment confounding			
		No^a^		Yes^b^	
		Biomarker +	Biomarker −	Biomarker +	Biomarker −
**No**	Proportion of patients	30%	70%	30%	70%
	Proportion receiving adjuvant treatment	80%	80%	90%	60%
	Treatment efficacy (HR)	0.6	0.6	0.6	0.6
	Event rate (baseline)	31%	15%	31%	15%
	Event rate (post adjuvant treatment)	**21%**	**10%**	**20%**	**12%**
	Sample size needed	~ 550		~ 1075 (2-fold)	
**Yes**	Proportion of patients	30%	70%	30%	70%
	Proportion receiving adjuvant treatment	80%	80%	90%	60%
	Treatment efficacy (HR)	**0.2**	**0.5**	**0.2**	**0.5**
	Event rate (baseline)	31%	15%	31%	15%
	Event rate (post adjuvant treatment)	**11%**	**9%**	9%*	11%^*^
	Sample size needed	~ 11650 (21-fold)		~ 11650 (21-fold)	

Modelling assumptions reported in this table are similar to UK/ANZ DCIS HER2 data and current use of adjuvant treatment in DCIS. BIOMARKER - Biomarker is expressed in 30% of samples and is associated with 2-fold increase in event risk. ^a^ No treatment confounding, for example, in a randomised trial OR when the biomarker (unlike HER2) is NOT associated with any features that influence adjuvant treatment selection (e.g. high grade, larger lesion size or necrosis which would normally lead to a greater use of adjuvant radiotherapy). ^b^ Treatment confounding present, for example, in a cohort study or single institution series when the biomarker (e.g. HER2) is associated with features that influence adjuvant treatment selection (e.g., high grade, larger lesion size or necrosis leading to a greater use of adjuvant radiotherapy in biomarker-positive subgroup even when biomarker status is not known). ^c^ Predictive interaction present (e.g. HER2), with a greater adjuvant treatment efficacy in biomarker-positive subgroup (HR = 0.2) as compared with biomarker-negative subgroup (HR = 0.5). ^*^ effect observed in the opposite direction of true effect.

HER2 overexpression in DCIS is associated with adverse histological features such as high grade, larger lesion size and necrosis. Therefore, unless adjuvant treatment is randomly allocated (i.e. randomised trial), HER2-positive DCIS is more likely to be treated with adjuvant radiotherapy than HER2-negative DCIS. Such treatment bias (without the knowledge of HER2 status) will result in more events being prevented in HER2-positive DCIS, and thus absolute event-rate difference between HER2-positive and negative subgroups will become smaller. Any study based on such real-life cohort will need doubling of sample size to be able to detect this difference. However, in the real world, the greater radiotherapy benefit in HER2-positive DCIS further worsens the lack of power, requiring a sample size at least one order larger in magnitude. The HER2-radiotherapy treatment interaction also presents a potential risk of observing a difference in the direction opposite to that of true difference; the study by Borgquist and colleagues [3] may be one such example. This underscores the underappreciated fact that evaluating a prognostic and predictive biomarker, like HER2, in datasets where adjuvant treatment is not randomly allocated is fraught with a high level of uncertainty. A case-control design can be used to evaluate the prognostic characteristics of a biomarker by using adjuvant treatment as a matching variable. This eliminates the treatment related confounding, for example, as done by Visser and colleagues [4] in the PRECISION consortium or by us in evaluating the prognostic role of ER [5]. However, it is worth pointing out that in a case-control design, the role of matching variable can never be investigated and therefore such studies are inherently unable to investigate any predictive characteristics. A study that aims to evaluate both prognostic and predictive characteristics of a biomarker must be based on a randomised trial as is the case with the UK/ANZ DCIS biomarker study.

This brings us to the next question of whether it is possible to generate robust data that externally validate our findings of HER2 being a prognostic as well as predictive biomarker in DCIS. This, however, is not a realistic prospect in the short or intermediate term. Among the DCIS RCTs, NSABP B-17 [6] and EORTC10853 [7] have not been successful in collecting biospecimens, whereas all participants in NSABP-B24 [6] and NSABP-B35 [8] trials received adjuvant radiotherapy and therefore these trials are not suitable for addressing this question. Approximately 70% of participants in the IBIS-II DCIS trial [9] received adjuvant radiotherapy, but this allocation was not random. The RTOG9804 trial [10] had only 52 events thus limiting the power even if biospecimen collection was to be successful. SweDCIS trial [11] offers the only realistic prospect, but commercial interests may mean that such validation may not happen.

In summary, UK/ANZ DCIS results demonstrating prognostic and predictive role of HER2 are the most robust results to date and will likely be the only reliable data for a foreseeable future. Clinical practice is full of examples (e.g. advent of various laparoscopic approaches) where the practice changed with less robust data even when the potential for harm was much greater. The worst-case scenario in implementing these results would be undertreatment of some DCIS patients, which needs to be looked at in the context that adjuvant treatment in DCIS does not result in a significant improvement in overall survival. In other words, the possibility of harm is practically non-existent. We call upon the oncology community to embrace these results to avoid overtreatment of DCIS patients.
